# The Psychobiology of Bereavement and Health: A Conceptual Review From the Perspective of Social Signal Transduction Theory of Depression

**DOI:** 10.3389/fpsyt.2020.565239

**Published:** 2020-12-03

**Authors:** Annina Seiler, Roland von Känel, George M. Slavich

**Affiliations:** ^1^Department of Consultation-Liaison Psychiatry and Psychosomatic Medicine, University Hospital Zurich and University of Zurich, Zurich, Switzerland; ^2^Cousins Center for Psychoneuroimmunology and Department of Psychiatry and Biobehavioral Sciences, University of California, Los Angeles, Los Angeles, CA, United States

**Keywords:** bereavement, interpersonal loss, life stress, inflammation, immune system, biological aging, health, disease

## Abstract

Losing a spouse is considered one of the most stressful life events a person can experience. Particularly in the immediate weeks and months after the loss, bereavement is associated with a significantly increased risk of morbidity and mortality. Despite an abundance of research aimed at identifying risk factors for adverse health outcomes following marital death, the mechanisms through which mental and physical health problems emerge following bereavement remain poorly understood. To address this issue, the present review examines several pathways that may link bereavement and health, including inflammation and immune dysregulation, genetic and epigenetic changes, gut microbiota activity, and biological aging. We then describe how these processes may be viewed from the perspective of the Social Signal Transduction Theory of Depression to provide a novel framework for understanding individual differences in long-term trajectories of adjustment to interpersonal loss. Finally, we discuss several avenues for future research on psychobiological mechanisms linking bereavement with mental and physical health outcomes.

## Introduction

Losing a spouse can be a very stressful life event that places individuals at risk for mental and physical health problems (1, 2). Particularly in the immediate weeks and months following spousal loss, bereavement is associated with increased risk of multimorbidity and mortality (2–5), including an elevation in inflammation-related health problems (6–10), cardiovascular disease (CVD) (8, 11–14), and some types of cancer (12). Despite a large body of research in the trauma literature attempting to identify risk factors for adverse health outcomes following spousal bereavement, however, the mechanisms through which mental and physical health problems emerge following interpersonal loss remain poorly understood.

Over the past 30 years, the field of psychoneuroimmunology has helped elucidate how different life stressors affect autonomic nervous system, neuroendocrine, and immune processes that could in turn be relevant for understanding the psychobiology of bereavement (15, 16). In particular, the past decade has produced a substantial body of knowledge shedding light on how specific types of stressors can trigger increases in inflammation (15, 17–21), which has in turn been linked with the development of numerous disease conditions, including autoimmune disorders, CVD, and some cancers, as well as mortality (12). Consequently, bereavement-related dysregulation in immune function may be one potential process that underlies the increased risk for morbidity and mortality seen in spousal bereaved individuals (22).

One strategy for better understanding the psychobiology of bereavement involves applying what we know about depression, which is also strongly precipitated by interpersonal loss. One model in particular, the Social Signal Transduction Theory of Depression (20), may be helpful for shedding light on how spousal loss and grief affect neural and immune processes that in turn structure risk for health problems following bereavement. In brief, this theory suggests that both early and later-life stress can promote neuro-inflammatory sensitivity to subsequently occurring stressors and thus heighten a person's vulnerability to physical and mental health problems across the lifespan. In the context of bereavement, this would occur if an individual with a history of past life stress exposure lost a terminally-ill spouse in adulthood after a sustained period of caregiving burden, in turn leading to mental and physical health problems that have an inflammatory basis.

Recently, Knowles et al. (23) published an excellent systematic review that focused on the link between bereavement and immune system functioning. The present review also examines links between bereavement and immune functioning but seeks to go beyond prior work by providing an integrated account of psychosocial, neural, immunologic, and genomic processes linking bereavement and health, as well as a description of how cumulative lifetime stress exposure may alter vulnerability to mental and physical health problems following spousal loss. To accomplish this goal, we conducted a PubMed literature search of all relevant studies published through October 2019 using the following key words: bereavement, mental health, physical health, psychobiology, stress, genetic, epigenetic, neuroendocrine, neuroimmune, inflammation, and immunity. To be considered for this review, articles had to be peer reviewed and written in English (see Supplementary Table 1). The psychometric instruments used in the eligible studies are summarized in Table 1.

**Table 1:** Assessment instruments used in the studies evaluated.

**Abbreviation**	**Full name**
ATQ-P	Automatic Thoughts Questionnaire-Positive version
CSS	Crisis Support Scale
CTQ	Childhood Trauma Questionnaire
ECR-SF	Experiences in Close Relationships Questionnaire-Short Form
GMRI	Grief and Meaning Reconstruction Inventory
GMS	Grief Measurement Scale
HAMD	Hamilton Anxiety and Depression Scale
HRSD	Hamilton Rating Scale for Depression
HTQ	Harvard Trauma Questionnaire-Part IV
ICG	Inventory of Complicated Grief
ICG-R	Inventory of Complicated Grief-Revised
IES	Impact of Event Scale
LOT	Life Orientation Test
MCMI-III	Millon Clinical Multiaxial Inventory-III
PSS	Perceived Stress Scale
PERI-A	Psychiatric Epidemiology Research Interview-Anxiety Scale
PERI-H	Psychiatric Epidemiology Research Interview-Helplessness-Hopelessness Scale
PFQ-2	Personal Feelings Questionnaire-2
PG-13	Prolonged Grief 13 Items
PSOM	Positive States of Mind
PSQI	Pittsburgh Sleep Quality Index
RPT	Relationship Profile Test
SADS-L	Lifetime Version of the Schedule for Affective Disorders and Schizophrenia
SCID-I	Structured Clinical Interview for DSM-IV Axis I Disorders
SCL-90	Symptom Checklist-90
SF-36	36-Item Short Form Health Survey
SSI	Beck Scale for Suicidal Ideation
STRAIN	Stress and Adversity Inventory
TIPI	Ten-Item Personality Inventory
TSC	Trauma Symptom Checklist
TLEQ	Traumatic Life Events Questionnaire
YES	Yale Evaluation of Suicidality scale

## Social Signal Transduction Theory of Depression

Stressful life events, especially those involving interpersonal loss, are known to activate several autonomic, neuroendocrine, and neuroimmune pathways that can lead to increased inflammatory activity (16), which, if sustained, can increase risk for inflammation-related physical and mental health problems (20, 24, 25). Indeed, dysregulation of the immune system is a critical processes involved in the pathophysiology of various diseases, including infections, autoimmune diseases, CVD, and some cancers (19, 26, 27). In addition, inflammatory activity can negatively affect mental health, and has been found to play a role in the development of anxiety disorders, post-traumatic stress disorder (PTSD), and depression (26). In this context, the cytokines interleukin (IL)-1β (IL-1β), IL-6, and tumor necrosis factor-α (TNF-α) have been shown to be both upregulated by interpersonal life stress and associated with poor health (17, 28–32).

One lingering question in this context has always been how exactly stressful experiences like interpersonal loss affect neurocognitive processes that in turn increase inflammation and inflammation-related disease risk. This important question is addressed by the Social Signal Transduction Theory of Depression (20, 33), which is a multilevel theory that describes neural, physiologic, molecular, and genomic pathways that link interpersonal stress exposure with internal biological processes that increase risk for depression and depression-related health problems. More specifically, this theory hypothesizes that cumulative lifetime stress exposure, which encompasses both acute negative life events (e.g., interpersonal loss) and chronic difficulties, can increase inflammatory activity through the activation of the autonomic nervous system (ANS), hypothalamic-pituitary-adrenal (HPA), and systemic inflammatory response. Although the temporary engagement of these systems is critical for survival during times of actual social or physical threat, sustained activation can occur that increase a person's vulnerability to physical and mental health problems that have an inflammatory component (20, 24, 25, 34, 35).

Consistent with this theory, research has shown that genetic factors, epigenetic processes, personality traits (e.g., neuroticism), and social environmental conditions during childhood and adolescence (e.g., social/financial stress, uncertainty, abuse, or neglect) play a role in shaping individuals' sensitivity to later-occurring adverse life events (25, 36). At the molecular level, epigenetic regulation of gene transcription can play an important role in helping individuals adapt to challenges posed by the external social environmental (37). However, stress-induced epigenetic changes can also lead to persistent increases in social stress-related physiological reactivity that last for months or years (38). In sum, therefore, social-environmental and genetic processes can independently and interactively affect the likelihood that a particular adverse life event will get converted into changes in gene expression that have the ability to influence health (38, 39).

A growing body of research is showing that the gut microbiota may also play an important role in shaping the activity of the enteric nervous system, ANS, neuroendocrine pathways, and immune system (40). This research has shown that the signaling pathways linking the central nervous system and gut are sensitive to social-environmental factors (41). Moreover, disruption of these systems can result in altered gastrointestinal function, HPA-axis activation, changes in immune responses, and, therefore, increased inflammation-related behavioral changes and disease susceptibility (42, 43).

**Figure 1:**
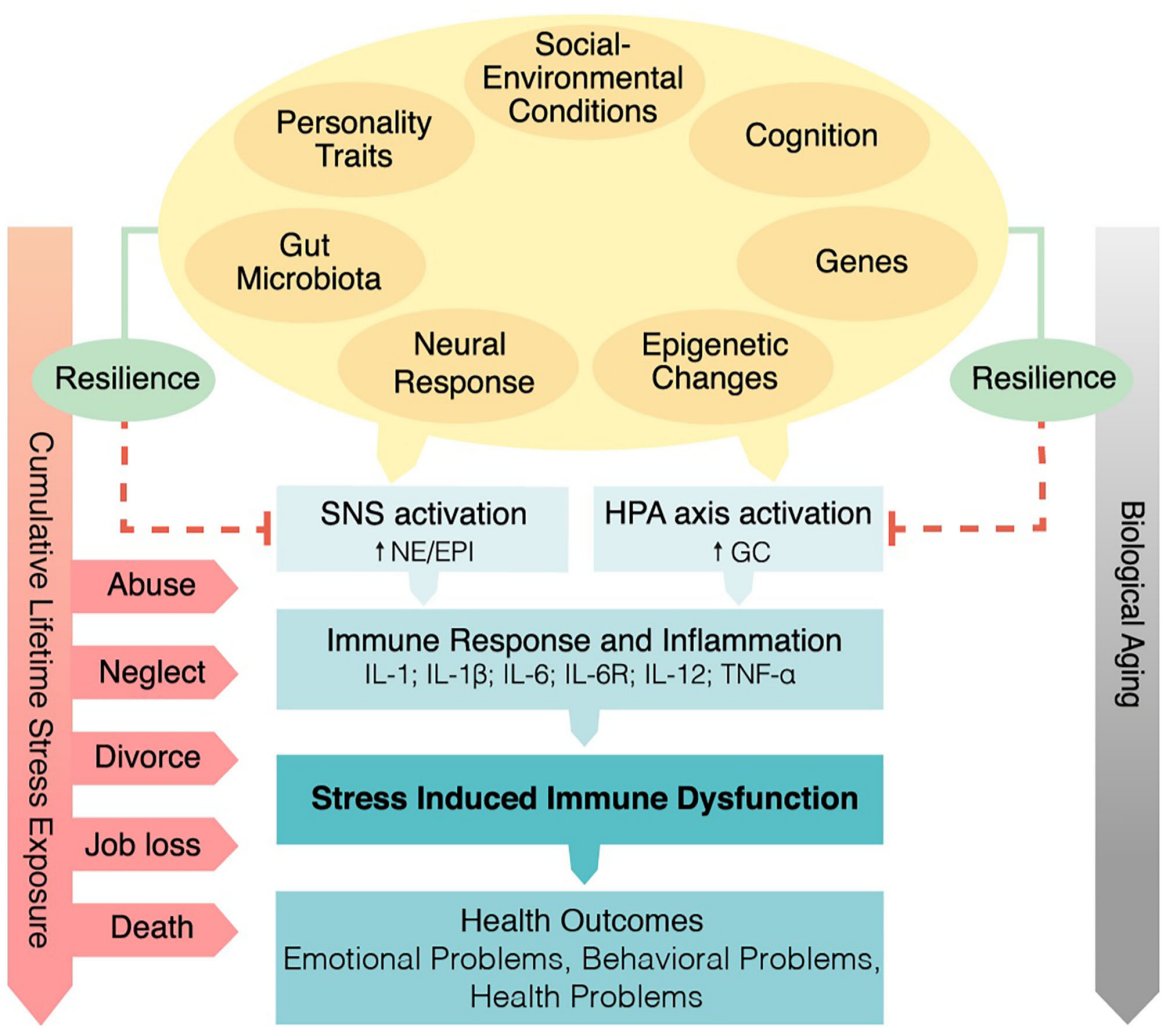
Psychobiological mechanisms linking spousal bereavement and health from the perspective of the Social Signal Transduction Theory of Depression (20). The interplay between genetic factors, personality traits (e.g., neuroticism), and social-environmental conditions during childhood and adolescence (e.g., social/financial stress, uncertainty, abuse, or neglect) are hypothesized to play important roles in shaping individuals' neuro-inflammatory sensitivity to later-occurring life stressors, including interpersonal loss. Exposure to early life stress and subsequent stressors are hypothesized to heighten sympathetic nervous system and hypothalamic-pituitary-adrenal axis dysregulation, and to promote increased inflammatory activity in response to a significant loss. If chronic, elevated inflammatory activity can in turn lead to a variety of adverse emotional, behavioral, and health outcomes. SNS, sympathetic nervous system activation; NE, norepinephrine; EPI, epinephrine; HPA, hypothalamic-pituitary-adrenal; GC, glucocorticoid; IL-1, interleukin-1; IL-1β, interleukin-1beta; IL-6, interleukin-6; IL-6R, interleukin-6 receptor; IL-12, interleukin-12; TNF-α, tumor necrosis factor-α.

In sum, the Social Signal Transduction Theory of Depression provides one illustration of how interpersonal loss can lead to specific changes in sympathetic nervous system (SNS) and HPA-axis activity that interact with genetic, personality, and social-environmental factors to promote immune dysregulation and increased inflammatory activity, especially in vulnerable individuals. These biological changes can in turn lead to depression-like behaviors, including anhedonia, helplessness, social withdrawal, and fatigue (26), which are characteristic of some bereaved individuals, thus making the Social Signal Transduction Theory of Depression a potentially useful framework for understanding psychosocial and biological aspects of bereavement (see Figure 1).

## Psychobiology of Bereavement from the Perspective of Social Signal Transduction Theory of Depression

For the purpose of this review, the term grief and bereavement have been conceptualized with slightly different meanings. Whereas, grief refers to a person's emotional response to a loss, bereavement refers to the time period when an individual experiences sadness, grief and mourning after a significant loss. Typically, bereavement is the period during which time the most intensive grieving occurs (44).

The loss of a significant person in one's life is a unique social stressor that requires an individual to adapt, which differentiates it from other stressors such as caregiving, a conflictual marital relationship, or unemployment (45). To better understand psychological adjustment to significant interpersonal loss, research has employed concepts from the literatures on attachment theory, cognitive processing, and resilience. Early life attachment has been proposed to impact relationships in adulthood (46). In this regard, the loss of a spouse represents an attachment stressor that inherently has physiological effects, as attachment stress evolutionarily served to maintain proximity between bonded pairs (23).

As a highly stressful event, the death of a significant person is known to trigger biological responses via several autonomic, neuroendocrine, and inflammatory pathways (22). These responses, either directly or via interaction with the social environment, can cause alterations in biological functioning that include the onset of chronic low-grade inflammation, which can increase risk for sickness behaviors, infections, mental and physical health problems, and premature mortality in vulnerable individuals (26, 47–49). Consistent with this thinking, several lines of evidence indicate that stressful life events are strongly associated with altered immune function and the development of depression, especially for persons living in a high-risk environment (e.g., trauma exposure) who possess a genetic predisposition to depression (20, 50–52). Moreover, clinical studies have shown that depressive symptoms are prevalent in widows and widowers during the first 2 years of bereavement, with a particular high risk for individuals with a history of depression (53, 54). To the extent that grief and depression are precipitated most strongly by the same types of major life stressors (i.e., sudden interpersonal loss), it may be possible to apply what we know about the psychobiology of depression (e.g., using the Social Signal Transduction Theory of Depression) to better understand the psychobiology of bereavement.

Importantly, the grief response depends on the nature and quality of the lost relationship (55). For example, the unintentional death of a spouse will be experienced differently than the death of a child, parent, or sibling, or the death of a close other by suicide (56). Furthermore, the unexpected death of a loved one may trigger different grief reactions than the loss of a spouse due to a terminal disease (56, 57). Hence, the psychobiology of bereavement may differ depending on the specific types of loss experienced (58). In the present article, therefore, we focus specifically on spousal bereavement as a particular type of life stressor.

In the following sections, we examine the applicability of the Social Signal Transduction Theory of Depression for studying psychobiological pathways that may link bereavement with multimorbidity and mortality. To do this, we review available research describing the impact of spousal loss on (a) mental and physical health, (b) inflammation and immune dysregulation, (c) genetic and epigenetic changes, (d) gut microbiota activity, and (e) biological aging. We also describe the effects of cumulative life stress exposure on health in bereaved individuals and explore psychobiological factors that have been linked to vulnerability and resilience to mental and physical health problems following significant interpersonal loss. Finally, we discuss how Social Signal Transduction Theory of Depression can be extended to bereavement and highlight several avenues of research that may be fruitful to pursue on this topic.

### The Impact of Bereavement on Mental Health

As alluded to previously, the death of spouse is one of the most stressful life events a person can experience (59). Spousal bereavement due to cancer, for example, is usually preceded by a long illness trajectory, which is associated with high distress due to the spouse's progressive health deterioration, anticipatory grief about the spouse's inevitable death, adoption of supportive responsibilities, financial stressors, and disruption of the caregiver's social and personal life (60). Moreover, partners of patients who have been suffering from an advanced illness have been found to experience more emotional distress and adjustment problems, as well as greater pain, fatigue, sleep problems, and depression (61–65).

There is considerable variability in how people respond to the loss of a spouse. For example, although the vast majority of conjugally bereaved individuals are relatively resilient and adjust adequately without professional support (66, 67), 10–20% of bereaved spouses develop intense, prolonged grief (67–69). In addition, substantial evidence suggests that high levels of traumatic grief, depression, and anxiety at 6 months post-loss predict the development of even more serious mental and physical health problems up to 25 months post-loss in spousal bereaved individuals, including suicidal ideation, cancer, and heart attacks (70).

Grief is a typical psychological and emotional reaction to losing a significant person that is characterized by symptoms of intense distress, anxiety, yearning, longing, and sadness, all of which usually subside over time (71). Although grieving symptoms are similar in some ways to symptoms of Major Depressive Disorder (MDD), as described by the Diagnostic and Statistical Manual of Mental Disorders (DSM-5), these two conditions are classified as distinct constructs (72, 73). Prolonged or persistent grief, by definition, is a debilitating condition following a significant loss that consists of persistent and pervasive longing for, or preoccupation with, the deceased person that persists for 6 months or longer. Moreover, it is characterized by its clinical features, including emotional pain (e.g., sadness, guilt, bitterness, anger), difficulty accepting the loss, emotional numbness, feeling that a part of one died, and difficulties in engaging in social or other activities (69). The diagnostic criteria for prolonged grief reactions are specified in the DSM-5 as Persistent Complex Bereavement Disorder (PCBD) (74) and in the International Statistical Classification of Diseases 11th Revision (ICD-11) as Prolonged Grief Disorder (PGD) (75, 76). PGD can be diagnosed at 6 months and PCBD at 12 months following the loss (77).

Bereavement is associated with short- and long-term health consequences. In one study that examined the early bereavement period (i.e., 6 months post-loss), prolonged grief was found in 12.3% of 56 bereaved adults between 20 and 50 years old who lost their spouse due to cancer (78). In another study of 132 adults, almost 30% of bereaved adults who lost a close relative to cancer met criteria for post-traumatic stress disorders 1 month post-loss (79). Over the long term, complicated grief was found to be present in 24.6% of 668 cancer caregivers 9 months post-loss (80), and 48% of 88 cancer caregivers who suffered from increased levels of bereavement-related distress 3–5 years after the loss (81).

As briefly noted above, high levels of prolonged grief symptoms have been associated with greater disability and can compromise psychological and physical functioning for years, resulting in comorbid health problems. These health problems include impaired sleep (82, 83), depression (9, 84–86), suicidal ideation and attempts (12, 87), and anxiety and PTSD (88, 89), as well as adverse health behaviors (12), prolonged sick leave, and increased health services and medication use (90–92). Furthermore, grief severity assessed from 3 to 6 months post-loss has been found to predict functional impairment, depressive symptoms, and impaired sleep up to 18 months following the loss (86, 93).

Of note, depression, and bereavement share considerable commonalities in terms of disease risk (e.g., CVD) and changes in immune function (2, 6, 11, 94–97). Some researchers have thus suggested that it is not interpersonal loss or bereavement but rather depression that is responsible for causing the increases in morbidity and mortality seen in bereaved individuals (9, 12, 49, 89, 98). On the other hand, though, research has shown that grief and depression are independently associated with health-damaging inflammation (99). Therefore, it may be the case that bereavement and depression are associated with similar but distinct patterns of inflammatory activity, and that interpersonal loss is a common precipitating stressful life event that may lead to both grief and depression.

### The Impact of Bereavement on Physical Health

Bereavement has also been found to increase individuals' risk for physical health problems and, especially, immune-related illnesses such as CVD and cancer, in addition to increased mortality risk, within the first 3 years following a major loss (12, 92). Indeed, CVD is a major cause of morbidity and mortality following bereavement (100). In terms of underlying mechanisms, strong evidence has linked bereavement-related stress with increased risk for CVD via chronic low-grade inflammation, as commonly measured by circulating IL-6 or C-reactive protein (CRP) (27, 101, 102). In addition, elevated levels of CRP have been shown to be a strong prognostic factor for atherosclerosis and cardiovascular events (103, 104).

In terms of mortality risk, a meta-analysis of 15 prospective cohort studies and 2,263,888 participants found that relative to their married peers, recently bereaved spouses had a 41% increased risk of dying within the first 6 months following bereavement, independent of age and gender (overall RR 1.41; 95% CI 1.26, 1.57) (105). Another meta-analysis of more than 500 million people found a 23% increased risk of mortality among widowers as compared to married individuals (HR 1.23; 95% CI 1.19, 1.28), with a relatively higher risk for men (increased risk: 27%) than women (increased risk: 15%) (3). This phenomenon of increased mortality risk for bereaved individuals, specifically within the first 6 months after the interpersonal loss, has been called the “broken-heart phenomenon” (106) or “widowhood effect” (105). Nowadays, these terms have also been used in the context of Takotsubo cardiomyopathy, an acute reversible heart failure syndrome mimicking acute myocardial infarction that is frequently triggered by emotional stress, including loss and grief (107, 108).

Depression and stress are important risk factor for developing CVD, and depression has been associated with poorer coronary outcomes (94). Correspondingly, the death of a significant person is known as a key psychosocial risk factor for CVD (11). Research has shown that as compared to non-bereaved individuals, bereaved individuals exhibit lower heart rate variability and higher heart rate, systolic blood pressure, von Willebrand factor, factor VIII, and platelet/granulocyte counts (6, 8, 13, 109, 110). These findings have direct clinical relevance, given that prothrombotic changes have been associated with greater risk of CVD and mortality (111, 112). Of note, the adverse effects of stress on cardiovascular and other outcomes are often potentiated by unhealthy behaviors, including poor dietary choices and sleep hygiene, inadequate physical activity, alcohol and/or tobacco consumption, and poor medication adherence (113), all of which are risk factors for CVD in their own right.

Regarding the implications of bereavement for cancer risk, stress hormones, especially glucocorticoids, adrenaline, and noradrenaline, have multiple known effects on human tumor biology (114, 115). Specifically, SNS activation via adrenergic- and glucocorticoid-mediated mechanisms can increase inflammatory activity and alter immune defense mechanisms against tumors with implications for tumor progression (116). Consistent with these pathways, a few studies have suggested that bereavement is associated with increased risk for cancer incidence. For example, a historical study conducted by Prigerson et al. (12) found that bereaved individuals suffering from high levels of grief had a significantly greater risk of developing cancer within 6–25 months post-loss than those with low levels of grief, yet the type of cancer was not described. Additionally, the Pan American Health Organization/World Health Organization examined the effects of parental bereavement on cancer incidence and survival (117). This large cohort study included 6,284 Jewish Israelis who lost an adult son in the Yom Kippur War or in an accident between 1970 and 1977, and who were followed longitudinally for 20 years. There was an increased incidence of lymphatic and hematopoietic malignancies in both war-bereaved parents (OR 1.47) and parents of accident victims (OR 2.01). There also was an increased incidence of melanoma among both war-bereaved parents (OR 1.72) and parents of an accident victim (OR 4.62). Finally, in parents suffering from cancer before the loss, bereavement was associated with a relatively shorter survival time.

These data highlight a possible association between bereavement and cancer. Consistent with this work, data from the psycho-oncology literature suggest that the incidence of cancer recurrence may be higher during bereavement-related distress (114–116, 118–122). On the whole, though, the available evidence linking bereavement and cancer risk is not highly consistent and is certainly limited relative to the relatively large amount of research demonstrating an association between bereavement and CVD risk.

Another condition associated with bereavement is type 2 diabetes, a chronic metabolic disorder characterized by insulin resistance due to insufficient insulin secretion and action (123). A growing body of research indicates that stress plays a role in type 2 diabetes, both as a predictor of new-onset type 2 diabetes and as a prognostic factor for individuals with existing type 2 diabetes. Stress-related biological pathways that are believed to contribute to the pathogenesis of type 2 diabetes include chronic activation of the HPA axis, which can result in neuroendocrine dysfunction and dysregulated cortisol output (123) that leads to glucose intolerance and systemic insulin resistance (124).

Only a limited number of studies have investigated the onset of type 2 diabetes in the context of bereavement. One study reported a 1.4-fold increased risk of incident diabetes in parents who lost their child relative to age- and sex-matched non-bereaved parents (125). Another study found that bereavement-induced prenatal stress in mother increased the risk of insulin resistance by 1.3-fold in their offspring; if grief was due to the death of an older sibling, the risk that the offspring developed type 2 diabetes during childhood or young adulthood was increased by 1.5-fold (126).

Taken together, these data provide relatively strong converging evidence that bereavement is associated with increased risk for poor mental and physical health. In most cases, the mechanisms underlying these bereavement-related health effects remain unclear. To address this issue, in the following sections we review psychoneuroimmunological pathways that may link bereavement with mental and physical health problems. We also discuss how these effects may be understood through the lens of the Social Signal Transduction Theory of Depression.

## Psychobiological Mechanisms Linking Bereavement and Health

### Bereavement-Induced Systemic Inflammation and Immune Dysregulation

#### Neuroendocrine, Sympathetic, and Inflammatory Activation

Several psychological, neural, and immunologic pathways may link bereavement with health. For example, it is well-known that stress activates the HPA axis and sympathetic-adrenal-medullary (SAM) axis, which in turn trigger the release of hormones that modulate immune function. Specific hormones involved in this stress-induced biological cascade include adrenocorticotropic hormone (ACTH), cortisol, growth hormone, prolactin, adrenaline, and noradrenaline (15). Through these mediators, life stress exposure can increase inflammatory activity and reduce anti-viral immune responses (17, 127).

In the context of bereavement, studies have begun to explore bereavement-related alterations in biomarkers of neuroendocrine and immune function. Results from these studies show that within the first 6 months following spousal loss, bereaved individuals exhibit evidence of reduced antibody response to vaccination (128), HPA-axis dysregulation [e.g., as indexed by higher cortisol levels and flatter diurnal cortisol slopes (9, 59, 129–132)], heightened systemic inflammation (6, 10, 49, 99, 133), and impaired immune responses, as indexed by reduced functional activity of natural killer cells (9, 130).

Research has also suggested that these bereavement-related biological alterations, particularly those involving inflammation, may underlie the development of complicated grief. For example, in a recent study of 99 spousally bereaved individuals by Fagundes et al. (99), bereaved individuals with greater grief severity and higher levels of depression showed higher levels of the pro-inflammatory cytokines interferon gamma (INF-γ), IL-6, and TNF-α approximately 3 months after the death of their spouse as compared to those who had more mild emotional reactions to the loss. Sleep disturbances can also induce inflammation (134). In a study of 54 spousally bereaved and 47 non-bereaved individuals, self-reported sleep disturbance in bereaved individuals was not directly related to elevated inflammatory activity, but bereavement moderated the association between sleep disturbances and inflammation approximately three moths post-loss, after adjusting for depression (133). Therefore, sleep disturbance may be an important pathway linking bereavement with increased inflammation and subsequent health problems.

Mechanistically speaking, the pro-inflammatory cytokines IL-1β, IL-6, and TNF-α are believed to influence the activity of neurotransmitters that can in turn affect mood and induce depressive symptoms in vulnerable individuals (28, 135). Consistent with this understanding, some studies have argued that changes in immune function and related neurochemical processes during bereavement are the result of bereavement-related depression as opposed to experiencing interpersonal loss or bereavement (136). Therefore, additional research is needed to examine the extent to which the increased inflammatory levels sometimes evident during or following bereavement are the result of an intense bereavement period itself vs. increases in depressive symptoms that sometime accompany bereavement.

#### Immune Dysregulation

Bereavement may also affect health by influencing immunity. Immunity is the natural or acquired resistance of an organism to bacterial or viral invaders, diseases, or infections, while having adequate tolerance to avoid allergy and autoimmune diseases (18). Lymphocytes, including T cells and B cells, as well as natural killer (NK) cells and macrophages, are the main types of cells of the immune system. T cells orchestrate the immune response via the production of cytokines and stimulate B cells to produce antibodies and signal killer cells to destroy antigen-displaying cells (137). Chronic stress, in turn, can induce low-grade inflammation and suppress immuno-protective cell function (18).

Studies investigating immune function in bereaved individuals have demonstrated NK cell activity suppression for up to 6 months following the sudden death of a relative (9, 130), lymphocyte proliferation for up to 2 months following spousal loss (138), and downregulated leukocyte gene expression in individuals who lost their spouse over the prior 2 years as compared to non-bereaved healthy adults (139). Seasonal influenza vaccination provides a useful paradigm to study individual differences in the inflammatory response. In one study that used this immunological challenge model in the context of bereavement, bereaved adults exhibited a reduced antibody titer response to influenza vaccination 1 year post-loss relative to non-bereaved adults (128). Together, these findings illustrate how bereavement can lead to long-lasting impairments in immunity that have implications for health.

### Bereavement-Induced Genetic and Epigenetic Changes

Another pathway by which bereavement may influence health is by affecting genetic and epigenetic pathways. Early life stress exposure has been associated with heightened physiological stress sensitivity as indexed by inflammatory activity and reactivity, as well as with greater immune system dysregulation later in life (30, 47, 140–143). In terms of gene expression, stress-induced epigenetic changes can result in altered gene expression (38).

The fields of psychoneuroimmunology, genetics, and genomics have only recently begun to examine genomic mechanisms that may underlie these effects. Although the literature examining bereavement-associated epigenetic changes remains scant, this research has already provided an interesting new window through which we may be able to better understand how bereavement affects health (34, 144). In this context, a few genetic polymorphisms have been investigated that could represent potential protective or risk factors in the face of adversity. For example, one recent cross-sectional study investigated the gene × environment (G × E) interaction effects of different genotypes and inflammation for 36 spousal bereaved older adults who experienced spousal loss over the past 23.75 months vs. 28 married individuals in order to elucidate factors predicting resilience during bereavement. The researchers found that spousally bereaved individuals exhibited higher levels of circulating inflammatory markers relative to non-bereaved individuals. Moreover, the authors identified a SNP in the IL-6-174 region that moderated individuals' vulnerability to increased systemic inflammation following the death of their spouse (49).

Relatedly, a cross-sectional study by O'Connor et al. (139) identified a genotype that helped explain the variability in grief severity experienced by older adults with and without complicated grief following the death of their partner or spouse. Individuals with both complicated grief (*N* = 12) and non-complicated grief (*N* = 24) exhibited the upregulated expression of genes implicated in the activation of the immune response and downregulated expression of genes implicated in B lymphocyte responses. Moreover, individuals exhibiting complicated grief showed a substantial downregulated expression of Type I interferon transcripts.

It is possible that mutations in these genes may lead to the altered regulation of pro- and anti-inflammatory cytokine gene expression, in turn placing individuals experiencing bereavement-related distress at increased risk of experiencing immune-related health problems following bereavement (26). Furthermore, changes in gene expression following bereavement may interact with epigenetic changes that have occurred earlier in life (e.g., from early life stress exposure) to influence how an individual responds to the loss of a spouse in later adulthood. Likewise, bereavement itself may cause epigenetic changes that could increase a person's risk of subsequent health problems through the activation of the previously described inflammatory pathways (34).

Together, these data suggest that increased inflammatory activity may predominantly occur in genetically susceptible individuals who experience the loss of a significant person. This G × E effect may provide a possible mechanism through which bereavement-related stressors increase vulnerability for morbidity and mortality in bereaved individuals. The G × E effect may also help explain why some bereaved individuals develop mental and physical health problems following the death of a spouse while others do not.

### Bereavement-Induced Changes in the Gut Microbiota

In addition, the gut microbiota may play a role in influencing health outcomes following bereavement. Evidence for this possibility comes from research documenting how acute and chronic stressors negatively impact the gut microbiome (41). Animal models provide experimental evidence for associations between early life stress exposure and low-grade inflammation, altered enteric microbiota, exaggerated stress reactivity, and visceral hypersensitivity (42, 145), emphasizing the impact of stress on microbial composition and activity. In humans, it is well-known that stress can cause nausea, vomiting, and abdominal pain (146). In addition, chronic life stressors such as financial problems, unemployment, and loss have been associated with an increased risk of developing functional gastrointestinal disorders, such as irritable bowel syndrome (147, 148). Moreover, accumulating evidence from clinical studies suggests a strong link between stress-induced disturbances along the gut microbiota-immune-brain axis and chronic inflammatory disorders, such as allergies, autoimmunity, and inflammatory gastrointestinal disorders (149, 150), and mental health problems including anxiety disorders and depression (151–155).

In contrast with this general body of research on stress and the gut microbiota, only a few studies to date have investigated how stress may lead to changes in the gut microbiota in the context of bereavement. Moreover, much of the research examining the effects of stress on the gut microbiota is still from animal model studies (43). Therefore, the American Gastroenterology Association and American Psychosomatic Society have encouraged efforts to characterize pathways linking stress and the gut microbiota-immune-brain axis in large, prospective, longitudinal cohort studies to better understand microbiome composition and microbial function in healthy and diseased individuals (156). Ultimately, it is possible that stress may change microbiota and vice versa via neuroendocrine (157), inflammatory (158), and behavioral (e.g., depression) pathways (154), but additional research is needed on this topic in general and especially in the context of bereavement.

### Bereavement and Biological Aging

Bereavement may also affect health through stress-induced accelerations in biological aging as indexed, for example, by telomere length. Telomeres are short DNA sequences that are located on the end of chromosomes. Telomeres protect chromosomes from degradation by forming a cap that provides chromosomal stability. Telomeres also regulate cellular replication and cellular lifespan (159). Of clinical importance, telomere length shortens with age (160), and extensive research has also shown that accelerated shortening of telomeres is associated with premature cell death, senescence, apoptosis, and carcinogenesis, in turn increasing morbidity and mortality (161). Furthermore, there is emerging evidence for an association of chronic stress with greater oxidative stress, lower telomerase activity, and shorter telomere length, indicating that stress may contribute to accelerated biological aging, thus providing a possible mechanism linking stress with health (39, 159, 162, 163).

Telomere attrition has emerged as a potentially useful biomarker of cellular aging that has shown associations with increased risk of various diseases and poorer survival (164). Additionally, the long-term effects of bereavement-related stress have been demonstrated in a study that investigated the impact of death or a sudden severe illness of a close family member during prenatal development. The study found that young adults with mothers who had experienced a death during prenatal development had shorter leukocyte telomere length as compared to young adults with mothers who had a pregnancy without experiencing death (165). These findings are of clinical importance for two reasons: first, telomere length in newborns and young adults has been related to maternal stress during pregnancy; and second, cellular aging can be influenced through prenatal stress, thereby potentially increasing an individual's susceptibility to infectious and autoimmune diseases in later life (166, 167).

Moreover, biological aging can affect the immune system by causing a progressive decline in functional immunity, referred to as *immunosenescence*, which diminishes humoral and cellular immune responses. Immunosenescence typically occurs in older individuals (≥65 years old) (168, 169). Declining T-cell function is a well-characterized feature of immunosenescence, contributing to chronic low-grade inflammation (170). However, chronic stress also has been shown to suppress and dysregulate immune function via immunosenescence (162). Consequently, age-associated declines in immune function can contribute to many comorbid conditions and may render older individuals more vulnerable to further assaults on their immune system from things like stress, immune-related illnesses, and infectious diseases (171). For instance, systemic low-grade inflammation, as indexed by circulating levels of CRP and IL-6, has been identified as a significant risk factor for CVD in elderly individuals (102).

The loss of a spouse or partner occurs most frequently in later life and is thus a life event that is often experienced by individuals older than 65 years, mainly due to the loss of a spouse (172). Several studies have found evidence of reduced immunity in older adults who have experienced bereavement. For instance, older bereaved vs. non-bereaved individuals with a mean age of 75 years have been found to show a weaker antibody response to vaccination in the year after the loss of a spouse (128). Furthermore, older bereaved individuals (*M*_age_ = 72 years old) have been found to exhibit impaired neutrophil function, lower neutrophil reactive oxygen species (ROS) production, and a higher cortisol-to-dehydroepiandrosterone sulfate (DHEAS) ratio relative to younger adults (*M*_age_ = 32 years old) (173, 174). Of note, cortisol is generally immunosuppressive, whereas DHEAS (secreted by the adrenal gland) is immune-enhancing and counterbalances the effects of cortisol on the innate immune system (175). With aging, though, cortisol and DHEAS respond differently to stress, resulting in a negative regulatory effect on immune function (173).

In addition, from a molecular perspective, older bereaved adults (aged 61–83 years old) have been found to show the reduced expression of genes involved in the B lymphocyte immune response as compared to a sample of age- and sex-matched non-bereaved adults (139). The salient incidence of immune-related dysregulation in older bereaved individuals suggests a pivotal role of mutually enhancing effects of stress and inflammation on immunosenescence (176). Indeed, bereavement-related distress may promote a natural decline of the immune system and increase older adults' risk of bereavement-related morbidity and mortality (177).

### Cumulative Life Stress Exposure and Bereavement

Chronic stress, especially when experienced in early life, can also affect health outcomes in bereaved individuals by setting the stage for long-lasting neurobiological changes that are associated with increased risk of later morbidity and mortality (178). Specifically, stress exposure during childhood can alter behavioral and physiological responses to acute and chronic stress in adulthood that in turn influence later-life risk for mental and physical health problems, including anxiety disorder, depression, CVD, and autoimmune and neurodegenerative disorders (20, 39, 47, 140, 179). Epigenetic changes and immune system dysregulation may be potential pathways that underlie this link (36, 39, 180). Indeed, the presence of multiple childhood adversities has been found to heighten emotional and physical reactivity to subsequent stress, which in turn activates genetic and epigenetic processes that may promote a proinflammatory phenotype (20).

In addition, cumulative life stress exposure is an important moderator of the association between acute life events, such as the loss of a spouse, and vulnerability to mental and physical health problems. As proposed by the Social Signal Transduction Theory of Depression, for example, greater lifetime stress exposure can increase neuro-inflammatory sensitization to adversity that increases a person's risk for immune-related mental and physical health problems in the face of subsequent interpersonal loss (20). Consistent with this formulation, among individuals with a history of childhood maltreatment, those who have experienced spousal loss have been found to be more likely to develop depressive symptoms than those who have not experienced such loss (84). These results suggest that adverse childhood experiences may set the stage for experiencing worse immune-related health outcomes following a significant loss in adulthood.

### Psychosocial Factors Affecting Risk and Resilience in Bereavement

Finally, several psychosocial factors can affect risk and resilience for poor health following bereavement. Resilience to stress is characterized by an individual's ability to maintain or restore relatively stable psychological and physical functioning when confronted with stressful life events, such as the death of a spouse (181). It has been hypothesized that resilience to such stressors arises from a combination of genetic factors (e.g., regulatory SNPs), personality traits (e.g., neuroticism, rejection sensitivity), and social-environmental conditions (e.g., life stress exposure, socioeconomic status) (182).

Bonanno et al. have conducted several seminal studies to better understand mechanisms underlying resilience (66, 181). For example, analyzing prospective longitudinal data from individuals enrolled prior to the death of a spouse, these investigators have identified four prototypical grieving trajectories: resilience (66.3%) and chronic depression (14.5%), followed by depressed improved (10.1%), and chronic grief (9.1%) (183, 184). Contrary to expectations, for most individuals (~60%), grief subsided over the initial weeks and months following the loss, indicating that most people are relatively resilient to interpersonal loss (184). However, even among resilient individuals, a majority showed grief reactions as characterized by transient distress, emotional pain, yearning, intrusive cognition, and rumination (185). In turn, only a small subgroup of grieving individuals exhibited severe grief that persisted for years after the marital loss (183, 184).

Psychosocial factors that appear to help buffer the negative effects associated with interpersonal loss include social support (186–188), secure attachment style (46, 189), positive emotions (129, 188, 190), optimism (191), cognitive flexibility (including positive reappraisal and acceptance) (192), and spirituality, including religiosity (193). In contrast, there are numerous psychosocial factors that have been found to weaken resilience to loss and grief, contributing to grief severity and prolonged grief reactions. These risk factors include the characteristics of spousal death [e.g., context of spousal illness, caregiving strain, lack of preparation for the death, traumatic loss (67, 194)], relationship quality [e.g., close kinship relationship, affection, intimacy, care, understanding, conflicts, ambivalence, and dependency in relationship (67, 195)], intrapersonal factors [e.g., extraversion, neuroticism, insecure, anxious or avoidant attachment styles, poor emotion regulation, negative cognition, pre-loss depression (67, 196, 197)], and interpersonal factors [e.g., low social and emotional support, financial hardships (67)].

In the bereavement literature, most studies that have been conducted so far have investigated group differences or correlates of bereavement, and only a few have examined biomarkers of resilience and vulnerability for bereavement-related morbidity. In this context, Buckley et al. (6) examined predictors of increased risk for thrombotic changes that might contribute to cardiovascular risk in the initial weeks following the death of a spouse and found a potential role for neutrophils, von Willebrand factor antigen, Factor VIII, and platelet/monocyte/granulocytes. Smoking was associated with a higher neutrophil count in acutely bereaved individuals and accounted for 6% of the shared variance (*r*^2^ = 0.06, *p* = 0.02). Moreover, duration of relationship between spousal bereaved and deceased spouse (*p* = 0.03) and smoking (*p* = 0.001) were found to be associated with higher platelet/granulocyte aggregate levels, accounting collectively for 14% of shared variance (*r*^2^ = 0.14).

In addition to this research, two studies have examined changes in immune function following spousal loss while differentiating between grief severity and depression. The first study by Fagundes et al. (99) found that grief severity and depression were independently associated with increased systemic inflammation (e.g., IL-6, TNF-α, IFN-y) in bereaved individuals approximately 3 months following the loss of a spouse. Furthermore, in the same study, MDD was a significant predictor of participants' levels of the proinflammatory cytokines IL-6, TNF-α, INF-γ, and IL-17A following spousal loss. Contrary to expectation, depression did not moderate the association between grief severity and inflammation. Likewise, a second study identified fatigue (as indexed by the SF-36 Energy/Fatigue sub-score) as a predictor of low-grade inflammation (as measured by CRP) in bereaved individuals 1 month following the loss of a spouse (10).

In general, these findings documenting psychosocial factors associated with risk and resilience in bereavement are preliminary and require replication to determine their reliability and relevance for predicting vulnerability to bereavement-related morbidity. Furthermore, most of the studies in this area have used cross-sectional, post-interpersonal loss research designs. Studies that include pre-loss measures of neuroendocrine and immune biomarkers, and that follow participants over time, are therefore needed.

## Discussion

### Bereavement From the Perspective of the Social Signal Transduction Theory of Depression

As discussed at the beginning of this review, we believe that psychobiological processes linking bereavement and health can be viewed from the perspective of the Social Signal Transduction Theory of Depression in order to provide one possible framework for better understanding processes that may underlie individual difference in risk and resilience to interpersonal loss. More specifically, the Social Signal Transduction Theory of Depression describes neural, physiologic, molecular, and genomic mechanisms linking interpersonal loss and health, as well as several moderators that can influence the effects of life stress on immune function and health, such as age, sex, and early life, adulthood, and cumulative lifetime stress exposure. Another important feature of the theory is that it differentiates between different types of life stressors and also accounts for individual characteristics—including both personality and genetic traits—that may render an individual differentially susceptible to the negative effects of stress and bereavement. Insights from this theory may thus help to address several existing questions in the bereavement literature, including why some individuals are more resilient than others to interpersonal loss, why cumulative life stress exposure is a strong predictor of morbidity and mortality in bereaved individuals, and why bereavement is associated with an increased risk of experiencing physical health problems. In light of this, we believe that identifying how social and biological factors identified by this theory might help explain differences in bereavement severity and persistence is an important topic for future research.

Integrating the consequences of the loss of a significant individual into the Social Signal Transduction Theory of Depression seems to be feasible with some modifications. For example, the Social Signal Transduction Theory of Depression emphasize that stressors experienced over time and exert a cumulative effect on neural and immunologic functioning that sensitizes a person to future stressors, such as interpersonal loss. In this model, the death of a loved one is regarded as a unique, complex stressor that can involve several types of adversity, including housing difficulties, financial strain, physical relocation, retirement, and the loss of daily routines and meaning in life (198, 199).

Relative to other topics in psychoneuroimmunology, the grief, loss, and bereavement literature has generated very few data documenting how lifetime stress exposure affects psychological, neural, and immunologic outcomes in bereaved individuals over time. It is possible that the severity or taboo of discussing grief has rendered the investigation of mental and physical health problems following spousal bereavement difficult. Moreover, obtaining funding for studies designed to examine health outcomes among bereaved individuals (as opposed to patients) can be difficult. As a result of these factors, despite the great importance of this topic, the health consequences of bereavement remain poorly understood (200, 201).

## Future Directions

To better understand how bereavement affects health, we believe there is a pressing need to study how bereavement leads to changes in psychological, neural, and immunologic functioning. Biobehavioral responses to grief, epigenetic changes, cumulative life stress exposure, and neural sensitivity to stress could each represent potential mechanisms linking bereavement and health. Therefore, it will be important to adopt a multidimensional approach to studying bereavement that involves assessing how these and other processes interact and change over time to structure differences in disease risk and longevity in the context of bereavement. Below, we discuss several direction for future research along these lines.

First, the early identification of individuals who are at the greatest risk of experiencing poor health outcomes following the death of a significant person is an important aim of precision medicine and disease prevention initiatives (202). Encouraging bereaved individuals to provide mental health and biological data will help advance research on how bereavement impacts mental and physical health. This knowledge can in turn help prevent or treat mental and physical health problems in bereaved individuals, especially those deemed to be at high risk as a function of their psychosocial or biological status. Ultimately, identifying biobehavioral mechanisms that can be modified or targeted early on in the bereavement process is an important and clinically relevant step toward the development of treatments that improve health outcomes in bereaved spouses.

Second, additional research is warranted to better understand the clinical significance, timing, duration, and trajectories of immune changes that occur following interpersonal loss (22). Large-scale longitudinal studies that systematically collect data on mental and physical health outcomes prior to and after spousal bereavement are needed. This longitudinal approach will help identify profiles that predict poor responses to the death of a significant person and thus aid in the identification of people who would benefit from personalized interventions and, moreover, the initial design of such interventions.

Third, studies investigating the biological bases of stress resilience will be important for elucidating processes that may help promote psychosocial resilience to interpersonal loss (203). From the perspective of the Social Signal Transduction Theory of Depression, vulnerability and resilience arises from a combination of social-environmental conditions (e.g., abuse or neglect, interpersonal, or financial difficulties), neurocognitive processes (e.g., perceptions of threat), social factors (e.g., social support), and genetics mechanisms (e.g., regulatory SNPs). According to this perspective, pre-loss neurocognitive, immunologic, and genetic functioning are important factors that help shape how a person is likely to respond to a significant interpersonal loss. Therefore, future studies examining psychobiological factors of resilience in bereavement could benefit from investigating G × E interaction effects by systematically assessing cumulative lifetime stress exposure (204) in addition to other predisposing psychosocial and biological factors that may confer increased vulnerability to the death of a significant person (38).

Fourth, it will be important to study health behaviors, such as smoking, diet, sleep, and exercise, which are not presently accounted for in the Social Signal Transduction Theory of Depression and that are rarely discussed in psychobiological models of bereavement. Some research has shown that bereavement is associated with negative health behaviors, especially diet and sleep (205, 206), but this literature is small for a topic that deserves serious attention.

Finally, investigating the interplay between multiple psychological and biological markers of stress over time involves substantial analytic computational complexity. Therefore, we believe that multilevel statistical approaches such as structural equation modeling and latent growth mixture modeling will be helpful for characterizing inter-individual differences in intra-individual changes over time (207).

## Conclusion

In conclusion, the death of a spouse is considered one of the most stressful life events a person can experience. In addition to increasing risk for depression, spousal death can lead to increased risk for a variety of somatic and physical diseases, as well as early mortality. Our goal with the present review was to help make sense out of these effects by reviewing social, psychological, immunologic, and genetic processes that have the potential to shape vulnerability to morbidity and mortality following spousal bereavement. We also related these processes to the Social Signal Transduction Theory of Depression, which we believe is one useful, multi-level framework that can be used to understand how social stressors affect psychobiological processes that impact health. In terms of future studies, it will be important to explore the associations described herein to help identify individuals at high risk for poor health outcomes following the death of a significant person. This knowledge could help to elucidate biobehavioral mechanisms that clinicians could in turn target early after a loss to improve health outcomes in bereaved spouses. Given the centrality of interpersonal loss to the human experience, we believe that much more research is needed to understand how exactly spousal bereavement affects health and how we can translate this knowledge to increase psychosocial resilience to such stress.

## Author Contributions

All authors developed the concept for this article. The initial draft was written by AS and subsequently edited by RK and GS. All authors read and approved the final version for publication.

## Conflict of Interest

The authors declare that the research was conducted in the absence of any commercial or financial relationships that could be construed as a potential conflict of interest. The reviewer LR declared a past co-authorship with one of the author GS to the handling editor.
