# Supplementary material for: The Psychobiology of Bereavement and Health: A Conceptual Review From the Perspective of Social Signal Transduction Theory of Depression
Source: Front Psychiatry. 2020 Dec 3;11:565239. doi: 10.3389/fpsyt.2020.565239 (PMC7744468; doi:10.3389/fpsyt.2020.565239)
Supplement: Supplementary file 1 [file Table_2_v1.DOCX]

Table 1. Summary of studies included in the review

| **Author, date** | **Title** | **Study design** | **Sample size** | **Outcome measures** | **Outcome** |
| --- | --- | --- | --- | --- | --- |
| Aalbaek et al., 2017 [14] | Risk of stroke after bereavement-a systematic literature review | Systematic literature review | 5 studies | To evaluate the association between bereavement and stroke | Bereavement-related stress is associated with a higher risk of stroke. |
| Barry et al., 2002 [89] | Psychiatric disorders among bereaved persons: the role of perceived circumstances of death and preparedness for death | Prospective study design, evaluation 4 and 9 months post-loss | 122 bereaved individuals | ICG-R  SCID-I for MDD and PTSD | Perception of the death as more violent was associated with major depressive disorder at baseline. Perception of lack of preparedness for the death was associated with complicated grief at baseline and at follow-up |
| Boelen et al., 2015 [193] | Optimism in prolonged grief and depression following loss: A three-wave longitudinal study | Prospective study design | 260 bereaved individuals | ATQ  SCL-90  LOT | Higher optimism at first year post-loss (T1) was associated with lower concurrent prolonged grief and depression severity. Higher optimism at T1 was also inversely related with depression symptom severity at 6 months (T2) and 18 months (T3), but not prolonged grief severity at T2 and T3. |
| Boelen et al., 2017 [78] | Disturbed grief: prolonged grief disorder and persistent complex bereavement disorder | Review article | - | - | When confronted with the death of a loved one, most people experience transient rather than persistent distress, and do not develop a mental health condition. PGD occurs in approximately 610% of bereaved individuals, with an in7creased risk following the death of a par8tner or child and loss to  unnatural or viole9nt circumstances, and among peopl10e vulnerable to mental health conditions. |
| Bonanno et al., 2002 [68] | Resilience to loss and chronic grief: a prospective study from preloss to 18-months postloss | Prospective study design | 205 individuals several years prior to the death of their spouse and at 6- and 18-months postloss | CES-D | Five core bereavement patterns were identified: common grief, chronic grief, chronic depression, improvement during bereavement, and resilience. Chronic grief was associated with pre-loss dependency and resilience with pre-loss acceptance of death and belief in a just world. |
| Bonanno et al., 2004 [68] | Prospective patterns of resilience and maladjustment during widowhood | Prospective cohort study | 1532 married individuals; 205 widow(er)s | CES-D  Self-developed coping variables, variables for meaning of the loss and context of the loss | Chronic grief stems from the upheaval surrounding the loss of a healthy spouse, whereas chronic depression results from more enduring emotional difficulties that are exacerbated by the loss. Both the resilient and the depressed-improved groups showed remarkably healthy profiles and relatively little evidence of either struggling with or denying/avoiding the loss. |
| Bonanno et al., 2005 [190] | Resilience to loss in bereaved spouses, bereaved parents, and bereaved gay men | Cross-sectional study design | 41 bereaved individuals  41 non-bereaved individuals | Structural clinical interviews | Resilience was evidenced in half of the bereaved sample when compared with matched, non-bereaved counterparts. Resilient individuals were not distinguished by the quality of their relationship with spouse/partner or caregiver burden but were rated more positively and as better adjusted by close friends. |
| Bottomley et al., 2015 [188] | Domains of Social Support That Predict Bereavement Distress Following Homicide Loss: Assessing Need and Satisfaction | Cross-sectional study design | 47 African Americans bereaved | Domains of social support | The griever’s level of satisfaction with physical assistance at the initial assessment best predicted lower levels of depression, anxiety, and posttraumatic stress disorder levels 6 months later, while less need for physical assistance predicted lower complicated grief at follow-up. |
| Buckley et al., 2012 [110] | Haemodynamic changes during early bereavement: potential contribution to increased cardiovascular risk | Prospective cohort study; evaluation at 2 weeks and 6 months following loss | 80 bereaved individuals and 80 non-bereaved | haemodynamic changes (blood pressure (BP) and heart rate (HR)) | The acutely bereaved had higher 24-hour systolic BP, higher daytime systolic BP, and higher daytime systolic load. Early bereavement is associated with increased systolic blood pressure and heart rate. These haemodynamic changes may contribute to a time-limited increase in cardiovascular risk. |
| Buckley et al., 2012 [13] | Effect of early bereavement on heart rate and heart rate variability | Prospective study design | 78 bereaved spouses and parents compared to no-bereaved individuals | Heart rate  Heart rate variability  CES-D | Acute bereavement was associated with increased 24-hour HR, reduced heart rate variability. At 6 months, the bereaved had a significantly lower heart rate. In the early weeks of bereavement, has demonstrated increased HR and altered autonomic function that might contribute to the increased cardiovascular events in early bereavement. |
| Buckley et al., 2012 [6] | Inflammatory and thrombotic changes in early bereavement: a prospective evaluation | Prospective study design 2 months and 6 months following bereavement | 80 bereaved spouses or parents  80 controls | Haemostatic measure  Neutrophil count  Willebrand factor antigen  platelet/granulocyte aggregates | Acute bereavement is associated with inflammatory and prothrombotic changes that may contribute to the increased cardiovascular risk with bereavement. |
| Carey et al., 2014 [11] | Increased risk of acute cardiovascular events after partner bereavement: a matched cohort study | Prospective study design (2005 – 2012) | N = 30’447 | Occurrence of a fatal or nonfatal myocardial infarction or stroke within 30 days of bereavement | 50 of the bereaved group (0.16%) experienced an MI or a stroke compared with 67 of the matched non-bereaved controls (0.08%) during the same period. The increased risk was seen in bereaved men and women and attenuated after 30 days. |
| Chen et al., 1999 [71] | Gender differences in the effects of bereavement-related psychological distress in health outcomes | Prospective study design with follow-ups at 6 weeks, 6, 13 and 25 month | 150 widow(er)s | CES-D  PERI-A/PERI-H  ICG | High symptom levels of traumatic grief measured at 6 months predicted a physical health event (e.g. cancer, heart attack) at 25 months post-intake for widows. High symptom levels of anxiety measured at 6 months predicted suicidal ideation at 25 months for widowers. |
| Chen et al., 2019 [85] | Differential psychological reactions to grief: The role of childhood adversity for depression symptoms among bereaved and non-bereaved adults | Cross-sectional study design | 44 bereaved individuals  44 controls | CTQ  CES-D | A simple slopes test indicated a positive association between childhood maltreatment and depressive symptoms among those who were bereaved (B = 0.86, p < .001), but such association did not emerge among those who were not bereaved (B = 0.06, p = .60). |
| Chirinos et al., 2018 [135] | Bereavement, Self-Reported Sleep Disturbances, and Inflammation: Results From Project HEART | Cross-sectional study design | 54 bereaved individuals and 47 controls | C-reactive protein  PSQI  CES-D | Sleep disturbances were not associated with elevated levels of C-reactive protein in the overall group. However, bereavement moderated the association between inflammation and sleep disturbances. |
| Chiu et al., 2010 [81] | Determinants of complicated grief in caregivers who cared for terminal cancer patients | Prospective study design | 668 bereaved caregivers | ICG | Female gender, spouse relationship, parents-children relationship, lack of religious belief, unavailable family support, and history of mood co-morbidity were risk factors that would predispose towards complicated grief. |
| Cohen et al. 2015 [7] | The association between bereavement and biomarkers of inflammation | 2^nd^ analyses of Midlife in the United States (MIDUS) | N = 529 participants | Levels of interleukin 6 (IL-6), C-reactive protein (CRP), soluble intercellular adhesion molecule-1 (sICAM-I), Soluble E-selectin (sE-selectin) and cortisol | Recent bereavements is associated with higher levels of inflammation, particularly among individuals with higher BMI and/or chronic health problems. |
| Fagundes et al., 2018 [8] | Spousal bereavement is associated with more pronounced ex vivo cytokine production and lower heart rate variability: Mechanisms underlying cardiovascular risk? | Cross-sectional study design | 32 recently bereaved individuals  33 controls | blood draw, EKG, and self-report questionnaires | Bereavement is associated with a more pronounced ex vivo cytokine production and lower HRV in a population that exclusively consisted of widows and widowers. |
| Fagundes et al., 2019 [100] | Grief, depressive symptoms, and inflammation in the spousally bereaved | Cross-sectional study design | 99 recently bereaved individuals | IFN-γ, IL-6, TNF-α, IL17-A, IL-2. | Bereaved individuals with a higher grief severity (using an established cut-score) had higher levels of the proinflammatory cytokines IFN-gamma, IL-6, and TNF-alpha than those with less grief severity. Those who experienced higher levels of depression exhibited elevated levels of proinflammatory cytokines compared with those who had lower levels of depression. |
| Galatzer et al., 2012 [185] | Beyond normality in the study of bereavement: heterogeneity in depression outcomes following loss in older adults | Longitudinal prospective study with assessment at 6, 18, and 48 months after the spouses' deaths. | 545 bereaved spouses | Trajectories of bereavement | We uncovered four discrete trajectories similar in shape and proportion to the previous analyses: Resilience (characterized by little or no depression; 66.3%), Chronic Grief (characterized by depression following loss, alleviated by 4 years post-loss; 9.1%), Pre-existing Chronic Depression (ongoing high pre- through post-loss depression; 14.5%), and Depressed-Improved (characterized by high pre-loss depression that decreases following loss; 10.1%). |
| Gerra et al., 2003 [9] | Long-term immune-endocrine effects of bereavement: relationships with anxiety levels and mood |  | 14 bereaved individuals  14 controls | HAMD  adrenocorticotropic and cortisol plasma concentrations, and non-suppression in response to dexamethasone | The immunological consequences of stress do not simply overlap with psychological and endocrine alterations, and are particularly severe and long-lasting in a subgroup of subjects, indicating the importance of individual variability in the capacity to cope with stress. |
| Hall et al., 1997 [83] | Intrusive thoughts and avoidance behaviors are associated with sleep disturbances in bereavement-related depression | Cross-sectional study design | 40 bereaved individuals with major depression | EEG  EMG  HRS  IES  SADS-L | Greater frequency of bereavement-related intrusive thoughts and avoidance behaviors was associated with longer sleep latency and lower delta sleep ratio. |
| Hart et al., 2007 [4] | Effect of conjugal bereavement on mortality of the bereaved spouse in participants of the Renfrew/Paisley Study | Prospective cohort study 1972 and 1976 | 4395 married couples | Causes of death to 31 March 2004 | Bereaved participants were at higher risk than non-bereaved participants of dying from any cause. Conjugal bereavement, in addition to existing risk factors, is related to mortality risk for major causes of death. |
| Huh et al., 2018 [198] | Attachment styles, grief responses, and the moderating role of coping strategies in parents bereaved by the Sewol ferry accident | Cross-sectional study design (x̄_time_ 18 months post-loss) | 81 Bereaved parents | ECR-SF  Brief COPE  ICG  PFQ-2 | Anxious attachment was associated with severe shame/guilt, and avoidant attachment correlated with complicated grief. Anxious attachment was positively associated with all types of coping strategies, and avoidant attachment was negatively related to problem- and emotion-focused coping. |
| Irwin et al., 1988 [132] | Plasma cortisol and natural killer cell activity during bereavement | Prospective study design | N = 28, of those were 9 bereaved | Natural killer (NK) cell activity | Bereaved women showed reduced NK activity and increased plasma cortisol levels as compared to controls. Anticipatory bereaved women also showed significant reductions in NK activity, but had levels of plasma cortisol comparable to those of controls. |
| Khanfer et al., 2011 [176] | Neutrophil function and cortisol:DHEAS ratio in bereaved older adults | Between-subject design | 24 bereaved (within 2 months) and 24 age- and sex-matched non-bereaved controls | Neutrophil function in elders | Neutrophil superoxide production was significantly reduced among the bereaved when challenged with E. coli. the bereaved group had a significantly higher cortisol:DHEAS ratio compared to controls. The stress of bereavement exaggerates the age-related decline in HPA axis and combines with immune ageing to further suppress immune function, which may help to the explain increased risk of infection in bereaved older adults. |
| Kaprio et al., 1987 [101] | Mortality after bereavement: a prospective study of 95,647 widowed persons | Prospective cohort study 1972 and 1976 | 95,647 widow(er)s | Standardized mortality ratios | The highest relative mortality risk was found immediately after bereavement. For all natural causes, mortality during the first week was over two-fold compared to expected rates. Mortality from suicides was greater than expected during the first years of widowhood. |
| Keyes et al., 2014 [58] | The burden of loss: unexpected death of a loved one and psychiatric disorders across the life course in a national study | Prospective cohort survey (National Epidemiologic Survey on Alcohol and Related Conditions) | N = 27,534 | Unexpected death  DSM -IV mood, anxiety and alcohol use disorders | The bereavement period is associated with elevated risk for the onset of multiple psychiatric disorders, consistently across the life course and coincident with the experience of the loved one's death. |
| Kristensen et al., 2012 [80] | Posttraumatic Stress Disorder After Bereavement: Early Psychological Sequelae of Losing a Close Relative Due to Terminal Cancer | Cross-sectional study design | 132 recently bereaved individuals | HTQ  CSS  TSC | One month after the loss, 29.5% of the subjects had clinical PTSD and an additional 26.2% reached a subclinical PTSD level. Negative affectivity, social support, and locus of control in relation to the loss predicted 57% of the variance in PTSD severity. |
| Latham et al., 2004 [88] | Suicidality and bereavement: complicated grief as psychiatric disorder presenting greatest risk for suicidality | Cross-sectional study design at 6 months post-loss | 309 bereaved adults | ICG-R  SSI  YES | Cross-sectionally, complicated grief (CG) was associated with a 6.58 times greater likelihood of "high suicidality" at baseline, and an 11.30 times greater risk of high suicidality at follow-up, after controlling for gender, race, major depressive disorder (MDD), posttraumatic stress disorder. Longitudinally, CG at baseline was associated with an 8.21 (95% CI: 2.49-27.0) times greater likelihood of high suicidality at follow-up, controlling for the above confounders (PTSD), and social support. |
| Lerdal et al., 2016 [84] | Sleep among bereaved caregivers of patients admitted to hospice: a 1-year longitudinal pilot study | Prospective study design | 16 bereaved family caregivers | PSQI | Results from this pilot study indicate that sleep problems are common for caregivers and continue into the bereavement period, particularly for partner caregivers. |
| Levav et al., 2000 [113] | Cancer incidence and survival following bereavement | Prospective cohort study with a follow-up of 20 years | N = 6284  Jewish Israelis who lost an adult son in the Yom Kippur War or in an accident between 1970 and 1977 | Cancer incidence in bereaved versus non-bereaved individuals | Increased incidence was found for lymphatic and hematopoietic malignancies as well as for melanomas among the parents of accident victims (odds ratio [OR] = 2.01) and among war-bereaved parents (OR = 1.47). The risk of death was increased by bereavement if the cancer had been diagnosed before the loss, but not after. |
| Lundorff et al., 2017 [69] | Prevalence of prolonged grief disorder in adult bereavement: A systematic review and meta-analysis | Meta-analysis | 14 eligible studies | To estimate the prevalence rate of prolonged grief disorder (PGD) in the adult bereaved population, identify possible moderators, and explore methodological quality of studies in this area | Meta-analysis revealed a pooled prevalence of PGD of 9.8%. Higher mean age to be associated with higher prevalence of PGD. |
| Maccallum et al., 2015 [186] | Trajectories of depression following spousal and child bereavement: A comparison of the heterogeneity in outcomes |  | 2512 bereaved adults | RPT  TIPI  The Continuing Bonds Scale  UCLA Loneliness Scale | Four discrete trajectories were identified: Resilience (little or no depression; 68.2%), Chronic Grief (an onset of depression following loss; 13.2%), Depressed-Improved (high pre-loss depression that decreased following loss; 11.2%), and Pre-existing Chronic Depression (high depression at all assessments; 7.4%). |
| Mancini et al., 2015 [199] | Predictors of Prolonged Grief, Resilience, and Recovery Among Bereaved Spouses | Prospective study designed with assessments at 1.5 to 3 years postloss | 115 bereaved spouses  74 married controls |  | Prolonged grief, when compared to resilience, was uniquely associated with maladaptive dependency traits, difficulty accessing positive memories of the deceased, and higher recalled marital adjustment. |
| Melhem et al, 2001 [90] | Comorbidity of axis I disorders in patients with traumatic grief | Cross-sectional study design | 23 bereaved individuals | ICG  SCID-I for DSM-IV | Most subjects who met criteria for complicate grief, met criteria for a current or lifetime Axis I diagnosis. 52% met criteria for current major depressive disorder, and 30% for current posttraumatic stress disorder. |
| Moon et al., 2011 [106] | Widowhood and mortality: a meta-analysis | Meta-analysis | N = 2,263,888 from 15 prospective cohort studies | Widowhood and mortality | Widowhood effect was not different for those aged younger than 65 years compared to those older than 65. There was a difference in the magnitude of the widowhood effect by gender; for women the RR was not statistically significantly different from the null (overall RR = 1.04, 95% CI: 1.00, 1.08), while it was for men. |
| Moskowitz et al., 2003 [192] | Do positive psychological states shed light on recovery from bereavement? Findings from a 3-year longitudinal study | Prospective study design | 86 bereaved gay spouses | CES-D  PSOM  Ways of coping | Although the patterns of post-bereavement depressive mood and positive psychological states were similar, some of their predictors differed. Both positive and negative psychological states should be included in studies of post-bereavement adjustment. |
| O’Connor et al., 2012 [134] | Diurnal cortisol in Complicated and Non-Complicated Grief: slope differences across the day | Cross-sectional study design | Women with Complicated Grief (n=12) from those with Non-Complicated Grief (n=12) | Cortisol slope | Women with Complicated Grief, who were bereaved in the past 18 months, had a flatter slope across the day, when controlling for education and body mass index. |
| O’Connor et al., 2014 [141] | Divergent gene expression responses to complicated grief and non-complicated grief | Cross-sectional study design | 36 spousally bereaved individuals (within the past 2 years)  27 non-bereaved controls | Leucocyte gene expression  PSS  ICG  IES | Compared to non-bereaved controls, bereavement (both Complicated Grief and Non-complicated Grief) was associated with upregulated expression of genes involved in general immunologic activation and a selective downregulation of genes involved in B lymphocyte responses. Complicated Grief and Non-complicated Grief differed markedly in their expression of Type I interferon-related transcripts, with Non-complicated Grief subjects showing substantial upregulation relative to non-bereaved controls and Complicated Grief subjects showing substantial downregulation. |
| Olsen et al., 2005 [127] | Hospitalization because of diabetes and bereavement: a national cohort study of parents who lost a child | Prospective study design, 18 years follow-up following loss | 15 bereaved versus 15 non-bereaved individuals | Rate of hospitalization because of Type 1 or Type 2 diabetes | Psychological stress may be a contributing cause of, or have prognostic importance for, both Type 1 and Type 2 diabetes, but a statistically significant association was only seen for Type 2 diabetes. |
| Ong et al., 2011 [131] | Spousal loss predicts alterations in diurnal cortisol activity through prospective changes in positive emotion | Prospective study design | 132 bereaved individuals | Salivary cortisol levels | Spousal bereavement was associated with lower levels of cortisol at wakeup and a flattening of the diurnal cortisol rhythm, independent of age, gender, education, extraversion, neuroticism, negative emotion, medication use, and smoking. |
| Phillips et al., 2006 [130] | Bereavement and marriage are associated with antibody response to influenza vaccination in the elderly | Prospective study design. Antibody response was assessed at baseline, 1 and 12 months following vaccination | N = 184 | Antibody response to the annual trivalent influenza vaccination | Bereavement in the year prior to vaccination was negatively associated with the 1-month response to the A/Panama and B/Shangdong strains. Being married and having higher marital satisfaction was also associated with higher peak responses to the A/Panama influenza strain at 1 month. |
| Prigerson et al., 1995 [87] | Complicated grief as a disorder distinct from bereavement-related depression and anxiety: a replication study | Cross-sectional study design with evaluation at 6 months after spousal death | 150 widowed individuals | Grief Measurement Scale | Symptoms of complicated grief were distinct from the symptoms of bereavement related depression and anxiety. |
| Prigerson et al., 1997 [12] | Traumatic grief as a risk factor for mental and physical morbidity | Prospective study design including 6 week, 6-, 13- and 25 month follow-up | N = 150 widows & widowers | Grief Measurement Scale | It is not the stress of bereavement, per se, that puts individuals at risk for long-term mental and physical health impairments and adverse health behaviors. Rather, it appears that psychiatric sequelae such as traumatic grief are of critical importance in determining which bereaved individuals will be at risk for long-term dysfunction. |
| Prigerson et al., 2009 [70] | Prolonged grief disorder: Psychometric validation of criteria proposed for DSM-V and ICD-11 | Prospective study design | 291 bereaved individuals | To determine the psychometric validity of criteria for prolonged grief disorder (PGD) to enhance the detection | The results support the psychometric validity of the criteria for PGD that we propose for inclusion in DSM-V and ICD-11. |
| Richardson et al., 2015 [133] | How Does Bereavement Get Under the Skin? The Effects of Late-Life Spousal Loss on Cortisol Levels | Prospective study design, assessment 6 and 18 months following bereavement | 263 bereaved individuals | Stress hormone cortisol | Bereaved spouses who reported prolonged forewarning of the death evidenced higher cortisol levels at W1 than those who did not experience prolonged forewarning. Bereaved women had higher cortisol levels than bereaved men at W1. |
| Schleifer et al., 1983 [140] | Suppression of lymphocyte stimulation following bereavement | Prospective study design | 12 spousally bereaved individuals | Lymphocyte function | Lymphocyte stimulation responses to phytohemagglutinin, concanavalin A, and pokeweed mitogen were significantly suppressed during the first two months following bereavement. No differences were found in total lymphocyte or B- or T-cell numbers. Suppressed immunity following the death of a spouse may be related to the increased morbidity and mortality associated with bereavement. |
| Schulz et al., 2001 [92] | Involvement in caregiving and adjustment to death of a spouse: findings from the caregiver health effects study | Prospective, population-based cohort study conducted between 1993 and 1998 | 129 bereaved spouses | CES-D  Antidepressant medication use  Health risk behavior, weight | Among individuals who are already strained prior to the death of their spouse, the death itself does not increase their level of distress. Instead, they show reductions in health risk behaviors. Among non-caregivers, losing one's spouse results in increased depression and weight loss. |
| Schultze-Florey et al., 2012 [50] | When grief makes you sick: bereavement induced systemic inflammation is a question of genotype | Cross-sectional study design | 36 widow(er)s  28 married individuals | pro-inflammatory cytokine single nucleotide polymorphisms  Inventory of Complicated Grief | Results suggest a possible mechanism for the increase in morbidity and mortality in the surviving spouse. Genetic variability interacts with an environmental stressor, leading to increased inflammatory markers in genetically susceptible subjects only. |
| Seiler et al., 2018 [10] | Impaired mental health and low-grade inflammation among fatigued bereaved individuals | Cross-sectional study design | 78 bereaved individuals | CRP TNF-a IL-6  PSS CES-D, PSQI, SF-36 energy/vitality SF-36 pain, SF-36 general | Fatigued bereaved individuals showed elevated systemic inflammation as measured by CRP in comparison to non-fatigued bereaved individuals. They were also more likely to report mental health problems that co-occur with fatigue in the context of immune activation. |
| Shor et al., 2012 [3] | Widowhood and mortality: a meta-analysis and meta-regression | Meta-analysis | 1377 mortality risks from 123 publications and data from > 500 million persons | Mortality risk | Compared with married people, widowers had a mean hazard ratio (HR) of 1.23 (95% confidence interval (CI), 1.19-1.28) among HRs adjusted for age and additional covariates. A significant interaction effect was found between gender and mean age, with HRs decreasing more rapidly for men than for women as age increased. |
| Stahl et al., 2016 [2] | Mortality After Bereavement: The Role of Cardiovascular Disease and Depression | Prospective population-based cohort study (Cardiovascular Health Study) | N_Bereaved_ = 593  N_Controls_ = 593 | CES-D | Bereavement decreased mortality in women with CVD and increased mortality in men without CVD. |
| Stahl et al., 2017 [208] | Design and Rationale for a Technology-based Healthy Lifestyle Intervention in Older Adults Grieving the Loss of a Spouse | Prospective study intervention at baseline, 3, 6 and 9 months | N not mentioned | Test intervention for feasibility | An intervention to prevent depression, anxiety, and/or complicated grief disorder(s) among adults 60 years and older who are grieving the recent loss (within 8 months) of a spouse or partner was tested in a phase 1 and phase 2 study. |
| Stroebe et al., 2007 [189] | Does social support help in bereavement? | Prospective study design with assessment at 6, 18 and 48 months after loss | 60 bereaved individuals | CES-D | Bereaved individuals receiving high support reported lower depressive levels than those who receive low support. Social support helps individuals to cope with the loss of a spouse. |
| Tomarken et al., 2012 [79] | Examining the role of trauma, personality, and meaning in young prolonged grievers | Cross-sectional study design | 56 bereaved spouses | PG-13  ICG  GMRI  TLEQ  MCMI-III | Elevated levels of prolonged grief were found in this population, suggesting a need for further research into young spousal grief. |
| Virk et al., 2012 [128] | Prenatal exposure to bereavement and type-2 diabetes: a Danish longitudinal population based study | Prospective study design | 45302 children exposed to maternal bereavement | Diagnosis of type-2 diabetes | Children exposed to bereavement during their prenatal life were more likely to have a type-2 diabetes diagnosis later in life. These findings were most pronounced when bereavement was caused by death of an elder child. The second trimester of pregnancy to be the most sensitive period of bereavement exposure |
| Vitlic et al., 2014 [175] | Bereavement reduces neutrophil oxidative burst only in older adults: role of the HPA axis and immunesenescence | Cross-sectional study design | 21 young bereaved individuals (x̄_age_ = 32) and matched controls  26 older bereaved individuals (x̄_age_ = 72 years) and matched controls | Neutrophil levels of the cortisol and dehydroepiandrosterone sulphate (DHEAS) | Young bereaved participants showed robust neutrophil function when compared to age-matched non-bereaved controls, and comparable stress hormone levels, while reduced neutrophil ROS production and raised stress hormone levels (cortisol:DHEAS ratio) were seen in the older bereaved group compared to their age-matched controls. |
| Walsh et al., 2002 [195] | Spiritual beliefs may affect outcome of bereavement: prospective study | Prospective study design with assessment at 1, 9 and14 months | 135 bereaved individuals | The Royal Free interview for religious and spiritual beliefs | People who profess stronger spiritual beliefs seem to resolve their grief more rapidly and completely after the death of a close person than do people with no spiritual beliefs. |
| Young et al., 1963 [107] | The mortality of widowers | Prospective study design 1957 - 1963 | 486 widowers | Mortality rate | Increased rates of mortality among bereaved individuals. The shock of widowhood might weaken the resistance to other causes of death, and not just to suicide. |
| Zisook et al., 1991 [54] | Depression through the first year after the death of a spouse | Prospective study design during the first 13 months following spousal death DSM-III-R criteria for depressive episodes | 350 widow(er)s | DSM-III-R criteria for depressive episodes | 24% of widows and widowers met criteria for depressive episodes at 2 months, 23% did so at 7 months, and 16% did so at 13 months. Depressive episodes are common after the death of a spouse. |
| Zisook et al., 1994 [55] | The spectrum of depressive phenomena after spousal bereavement | Prospective study design with evaluation at 2, 13, and 25 months after the death of their spouse | 350 widow(er)s | DSM-III-R criteria for depressive episodes | Subsyndromal symptomatic depression prevalent, often persist, and are associated with substantial morbidity in widows and widowers during the first 2 years of bereavement. |
